# Which physical and social environmental factors are most important for adolescents’ cycling for transport? An experimental study using manipulated photographs

**DOI:** 10.1186/s12966-017-0566-z

**Published:** 2017-08-17

**Authors:** Hannah Verhoeven, Ariane Ghekiere, Jelle Van Cauwenberg, Delfien Van Dyck, Ilse De Bourdeaudhuij, Peter Clarys, Benedicte Deforche

**Affiliations:** 1 Department of Public Health, Faculty of Medicine and Health Sciences, Ghent University, De Pintelaan 185, B-9000 Ghent, Belgium; 2 Physical Activity, Nutrition and Health Research Unit, Faculty of Physical Education and Physical Therapy, Vrije Universiteit Brussel, Pleinlaan 2, B-1050 Brussels, Belgium; 3 Research Foundation - Flanders (FWO), Egmontstraat 5, B-1000 Brussels, Belgium; 4 Department of Movement and Sport Sciences, Faculty of Medicine and Health Sciences, Ghent University, Watersportlaan 2, B-9000 Ghent, Belgium

**Keywords:** Youth, Micro-environmental factors, Distance, Co-participation in cycling, Physical environment, Social environment, Experiment, Active transport

## Abstract

**Background:**

Ecological models emphasize that cycling for transport is determined by an interplay between individual, physical and social environmental factors. The current study investigated (a) which physical and social environmental factors determine adolescents’ preferences towards cycling for transport and (b) which individual, physical and social environmental factors are associated with their intention to actually cycle for transport.

**Methods:**

An online questionnaire consisting of questions on individual and social environmental variables, and 15 choice-based conjoint tasks with manipulated photographs was completed by 882 adolescents (55.3% male; 13.9 ± 1.6 years). Within the choice tasks, participants were asked to indicate which of two situations they would prefer to cycle to a friend’s house. The manipulated photographs were all modified versions of one semi-urban street which differed in the following physical micro-environmental attributes (separation of cycle path, evenness of cycle path, speed limit, speed bump, traffic density, amount of vegetation and maintenance). In addition, each photograph was accompanied by two sentences which described varying cycling distances and co-participation in cycling (i.e. cycling alone or with a friend). After each choice task participants were also asked if they would actually cycle in that situation in real life (i.e. intention). Hierarchical Bayes analyses were performed to calculate relative importances and part-worth utilities of environmental attributes. Logistic regression analyses were performed to investigate which individual, physical and social environmental factors were associated with adolescents’ intention to actually cycle for transport.

**Results:**

Adolescents’ preference to cycle for transport was predominantly determined by separation of cycle path, followed by shorter cycling distance and co-participation in cycling. Higher preferences were observed for a separation between the cycle path and motorized traffic by means of a hedge versus a curb, versus a marked line. Similar findings were observed for intention to cycle. Furthermore, evenness of the cycle path and general maintenance of the street were also of considerable importance among adolescents, but to a lesser extent.

**Conclusions:**

Results of this experimental study justify investment by local governments in well-separated cycling infrastructure, which seemed to be more important than cycling distance and the social environment.

## Background

Most adolescents do not achieve the recommended 60 min of moderate- to vigorous-intensity physical activity a day [1]. Active transport (e.g. walking and cycling) has great potential to increase adolescents’ physical activity levels since it can be easily integrated into daily routine [2–4]. In addition, active transport provides numerous benefits to the environment and public health [5]. Flanders (northern part of Belgium) is a walking- and cycling-friendly region characterized by good geographical and climatological conditions with adequate infrastructure and facilities to support active transport [6, 7]. A study among Flemish adolescents [6] showed that 58.4% of the sample commuted actively to school. However, nearly half of the passive commuters in that sample lived within a feasible active commuting distance from school. In contrast to other countries where higher walking rates were reported [8, 9], Flemish active commuting adolescents seem to cycle more frequently [6].

Ecological models emphasize that health behaviours, such as cycling for transport, are determined by an interplay between physical environmental factors (such as cycle path characteristics and distance), social environmental factors (such as co-participation in cycling, social modelling and social norms) and individual factors (such as gender, age and self-efficacy) [10]. Previous studies investigating physical environmental factors in relation to active transport mainly focused on macro-environmental characteristics such as residential density, land use mix and street connectivity, and found that a higher residential density, higher land use mix and higher street connectivity are positively associated with walking or cycling for transport [11–14]. Shorter distance has been found to be an important macro-environmental factor influencing adolescents’ cycling for transport in several studies [8, 15–19]. Physical macro-environmental factors are more difficult and expensive to change in existing neighbourhoods compared to physical micro-environmental factors (e.g. cycle path characteristics, vegetation and maintenance of the street). Studies investigating the association between physical micro-environmental characteristics and adolescents’ active transport in this age group are scarce and findings are inconsistent [11, 12, 19–21]. Kerr et al. [12] and Dalton et al. [20] found a positive association between the presence of sidewalks or cycle paths and US adolescents’ active transport levels, whereas Mota et al. [11] found no significant association among Portuguese adolescent girls. Furthermore, some studies [20, 21] found a positive association between the presence of trees and active commuting levels among adolescents.

Most studies investigating the association between the physical environment and adolescents’ cycling for transport used self-reported questionnaires to assess physical environmental factors [6, 12, 22, 23]. In order to tackle limitations of questionnaire-based studies (e.g. recall bias and difficulties to correctly define a ‘local neighbourhood’ [19, 24–26]), the current study used manipulated photographs in an experimental setting to investigate which physical micro-environmental factors are most important for adolescents’ preferences towards cycling for transport. Seven physical micro-environmental factors were manipulated in the photographs resulting in different street settings. Participants had to complete 15 choice tasks in which they were asked to indicate which situation they would most prefer to cycle to a friend’s house. Manipulated photographs do not require participants to recall an environment, and their experiences or perceptions since exposure to and assessment of the environment occurs simultaneously. Furthermore, there is no need to define a ‘local neighbourhood’ since the neighbourhood is presented in a photograph. In addition, manipulated photographs allow several physical micro-environmental factors to co-occur in one photograph which is consistent with most real-life situations. Using manipulated photographs is a good alternative to simulate potential changes to the micro-environment under controlled conditions, relatively quickly and at low cost. The use of manipulated photographs has been tested in several pilot studies [27–31] and has been successfully used to study the relationship between physical micro-environmental factors and a street’s appeal for active transport in other age groups [32–34]. However, manipulated photographs have not been used to investigate the relationship between physical micro-environmental factors and adolescents’ preferences towards cycling for transport.

A limitation of previous studies using manipulated photographs in other age groups was that distance to destination and the social environment were not taken into account. However, in adolescents, distance to destination and the social environment (e.g. cycling together with a friend) seem to play an important role in their choice to cycle for transport [8, 19, 22, 35–37]. Especially in this age group, the opinions and actions of peers strongly influence their own behaviour [38]. In a qualitative study [37], Flemish adolescents mentioned that they preferred to cycle for transport together with one or more friends. However, it is not clear whether distance to destination and social environmental factors are more important for adolescents’ cycling for transport than physical micro-environmental factors. In the current study, cycling distance and the social environment were included as extra experimental factors next to physical micro-environmental factors in order to gain insight into the importance of physical micro-environmental factors relative to cycling distance and social environmental factors. Another limitation of previous studies using manipulated photographs in other age groups [32–34] was that only the relationship between physical micro-environmental factors and a street’s appeal for walking or cycling was investigated. Although it is of considerable importance to investigate which factors determine adolescents’ preferences towards cycling for transport, it is also essential to know if they have the intention to actually cycle in the preferred situation. The Theory of Planned Behaviour, for example, emphasizes that intention of an individual to perform a given behaviour is the most proximal determinant of that behaviour [39].

The first aim of this study was to examine the importance of physical and social environmental factors regarding adolescents’ preferences towards cycling for transport using manipulated photographs. Secondly, our aim was to investigate which individual, physical and social environmental factors determine adolescents’ intention to actually cycle for transport in the preferred situation.

## Methods

### Protocol and participants

Adolescents aged 12–16 years (1st-4th year of secondary school) were recruited via randomly selected secondary schools across Flanders to participate in the study. A total of 103 secondary schools were contacted, of which 12 agreed to participate. The main reason why schools did not participate was because of their busy schedule. Within participating schools, a total of 1078 adolescents were invited to participate in the study. Passive informed consent was obtained from adolescents’ parents. If parents did not agree to let their child participate, they had to sign a form. Furthermore, researchers also obtained active informed consent of adolescents. A total of 1013 adolescents participated in the study (response rate = 94.0%) which was conducted at school under supervision of a researcher. School visits were conducted from March till October 2016. The study protocol was approved by the Ethics Committee of the Ghent University Hospital (2016/0285).

### Development of manipulated photographs

A computerized structured online questionnaire including choice-based conjoint tasks with manipulated photographs was developed using Sawtooth Software (SSI Web version 8.4.8). The photographs were all modified versions of one ‘basic’ panoramic photograph representing a typical semi-urban street in Flanders where adolescents could cycle. In order to standardize the photographs, the general street setting (i.e. typical semi-urban street), number of cyclists in the street and weather conditions were kept constant across all photographs. All photographs showed a cyclist’s point of view to create the feeling that one is cycling in the street. Seven physical micro-environmental factors (separation of cycle path, evenness of cycle path, speed limit, speed bump, traffic density, amount of vegetation and maintenance) were included in each photograph. Each factor consisted of at least two levels. A set of 1945 manipulated panoramic photographs, developed with Adobe Photoshop® software, was obtained. The selection of physical micro-environmental factors was based on existing literature in adolescents [11, 12, 19–21] and on previous research with manipulated panoramic photographs [28, 32, 40] studying relationships between the environment and cycling for transport among children and adults. An overview of included physical micro-environmental factors and their corresponding levels can be found in Table 1.

**Table 1:** Overview of included physical and social environmental factors and their corresponding levels

Factor	Level
Physical micro-environment	
Separation of cycle path	No cycle path
	Cycle path separated from traffic with lines, not separated from
	walking path (advisory cycle path)
	Cycle path separated from traffic with a curb, not separated from
	walking path
	Cycle path separated from traffic with a hedge, not separated
	from walking path
	Cycle path separated from traffic with a curb, cycle path different
	colour from walking path
	Cycle path separated from traffic with a hedge, cycle path different
	colour from walking path
Evenness of cycle path	Very uneven
	Moderately uneven
	Even
Speed limit	50 km/h
	30 km/h
Speed bump	Absent
	Present
Traffic density	4 cars + truck
	3 cars
	1 car
Amount of vegetation	No trees
	Two trees
	Four trees
Maintenance	Poor upkeep (much graffiti and litter)
	Moderate upkeep (a bit of graffiti and litter)
	Good upkeep (no graffiti or litter)
Physical macro-environment	
Cycling distance	Via this route it takes 15 min to reach your destination by bike
	Via this route it takes 14 min to reach your destination by bike
	Via this route it takes 13 min to reach your destination by bike
	Via this route it takes 12 min to reach your destination by bike
	Via this route it takes 11 min to reach your destination by bike
	Via this route it takes 10 min to reach your destination by bike
Social environment	
Co-participation in cycling	Via this route you will cycle alone
	Via this route you can cycle along with a friend

**Fig. 1:**
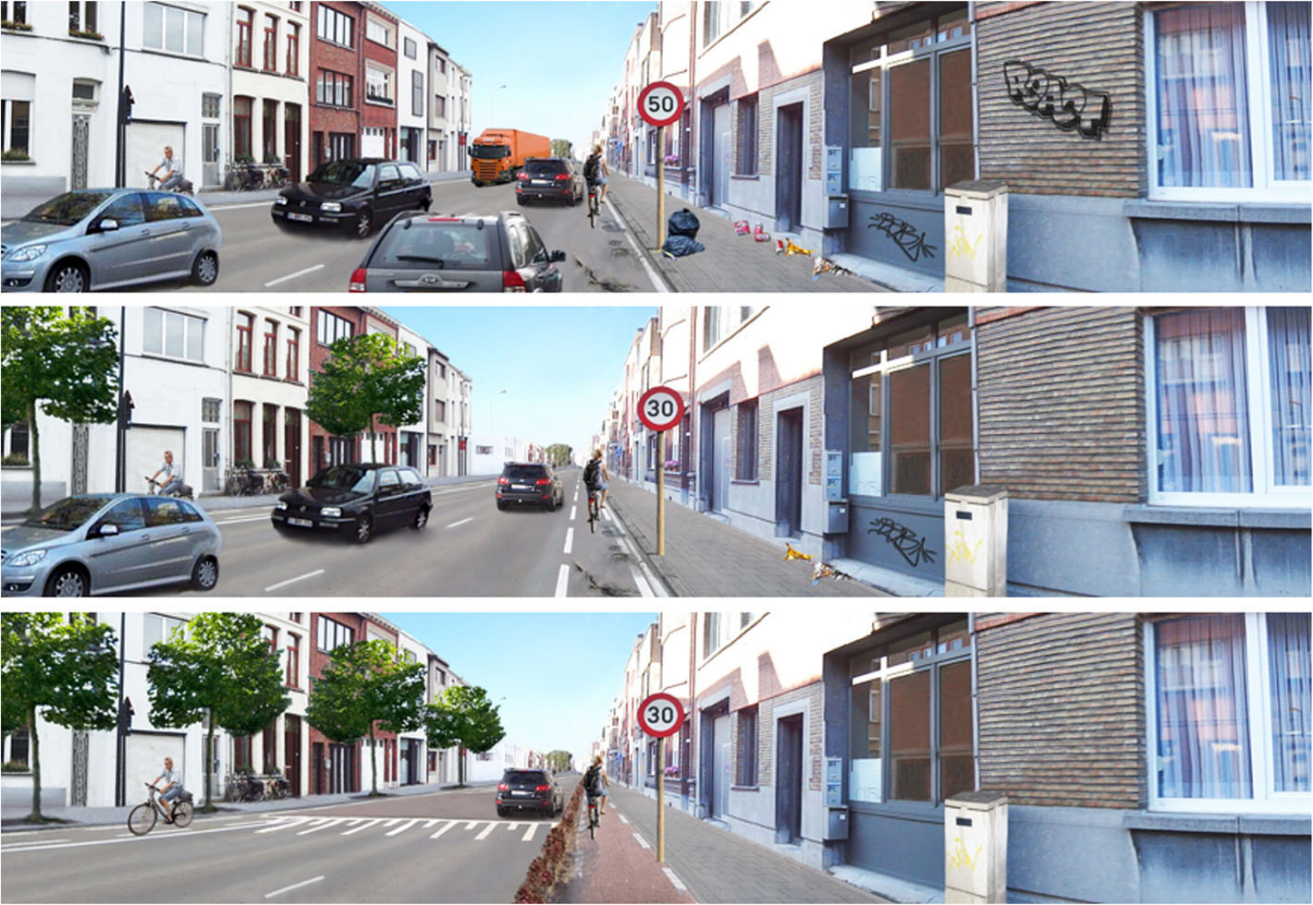
Examples of the manipulated photographs with the anticipated worst, medium and best setting to cycle

An example of the performed manipulations is shown in Fig. 1. The first photograph shows the anticipated worst setting to cycle along and the last photograph shows the anticipated best setting to cycle.

### Measures

Participants were asked to complete an online questionnaire including 15 choice-based conjoint tasks with manipulated photographs and questions on individual and social environmental factors.

#### Choice-based conjoint tasks

**Fig. 2:**
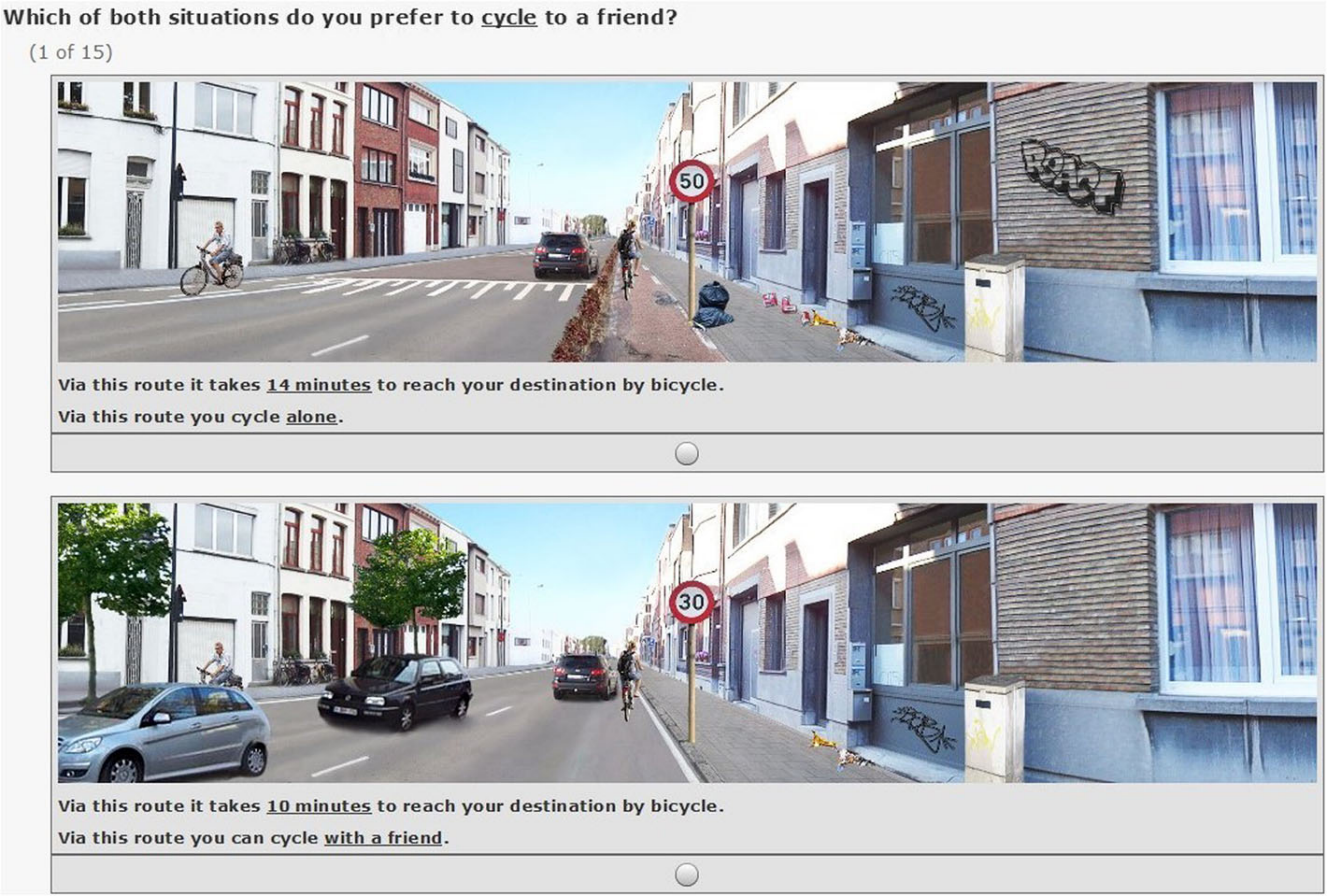
Example of a choice-based conjoint task

Adolescents completed a set of 15 choice-based conjoint tasks in which participants were asked to choose between two possible routes to cycle. Participants were asked to indicate which situation they would most prefer to cycle to a friend’s house. This choice-based conjoint method is often used in marketing research and aims to identify the relative importance of various components of a product in the decision process to pursue the product [41]. In the current study, the ‘products’ are manipulated photographs/street settings accompanied by two sentences which described varying cycling distances and co-participation in cycling (i.e. cycling alone or with a friend). The selection of cycling distance and co-participation in cycling was based on existing literature in adolescents [8, 15, 22, 38]. For cycling distance, six levels were included and for co-participation in cycling two levels were included. An overview of these factors and their corresponding levels can be found in Table 1. The research team chose to include cycling distances, operationalized by duration, between 10 and 15 min, with equal intervals of one minute since previous research showed that these are feasible cycling distances/durations for adolescents [6]. At the start of the choice tasks, the following standardized instruction was provided: “*Imagine yourself cycling to a friend’s house on the weekend during the daytime. The weather is ideal to cycle, it is not too warm, not too cold, there is no wind and it is not raining. Two photographs will appear displaying two different situations. The purpose is that you pick the situation which is the most attractive for you to cycle to a friend’s house. Please also pay attention to the (short) sentences underneath each photograph. There is no good or bad solution, we are just interested in what you consider most important while cycling to your friend’s house. A total of 15 combinations of situations will be presented to you. Please indicate for each combination which situation you prefer to cycle to a friend’s house.*” The choice tasks were full-profile, which implies that the two situations presented in one task could differ in one to nine attributes (seven physical micro-environmental factors, cycling distance and co-participation in cycling) [41]. Of the 15 choice-based conjoint tasks, 12 were random and three were fixed tasks. The 12 random tasks were different for all participants and were randomly assigned by the software. The three fixed tasks were similar for all participants and two of these tasks were identical to enable examination of test-retest reliability. After each of the 15 choice tasks, participants were asked the following question: *“In real life, would you actually cycle to a friend’s house in the situation you chose?”*. Answer options were as follows: *“Yes, I would cycle.”* and *“No, I would not cycle but choose another transport mode.”* An example of a choice-based conjoint task is shown in Fig. 2.

#### Individual factors

Socio-demographic information (e.g. school, study year, educational type, gender, age, nationality, living environment, education father, education mother), bicycle ownership, transport behaviour and self-efficacy towards cycling were also assessed. Education of parents was used to assess SES. Participants with both parents who completed only primary or secondary education were classified as lower SES and participants with at least one parent who completed tertiary education were classified as higher SES. Questions on transport behaviour were derived from the validated International Physical Activity Questionnaire (IPAQ) [42, 43]. Participants were asked to report frequency (days/week) and average daily duration of cycling to various destinations within the last seven days. Weekly minutes were calculated by multiplying frequency and duration of trips. Self-efficacy (1 item) was assessed on a five-point Likert scale by asking participants how confident they would be to cycle to a destination 10-min cycling distance from their home in potentially difficult situations (i.e. bad weather, when tired).

#### Social environmental factors

Finally, the following social environmental variables towards actual cycling were assessed: social modelling, social support and social norms. Social modelling was assessed by asking how frequently significant others (4 items: parents, siblings, friends, classmates) cycle to a destination. To investigate social support, participants were asked how often significant others (4 items) encourage them to cycle to a destination 10-min cycling distance from their home. Social norms were measured by asking if participants believed that significant others (4 items) wanted them to cycle for transport. For these variables, averages of item scores were used for data analyses. For all questions a five-point Likert scale was used and questions were based on existing questionnaires [22, 44].

### Analyses

Sample size calculations within Sawtooth Software showed that when including nine attributes and their corresponding levels, 270 participants were needed to obtain sufficient statistical power [45]. However, the present study was nested within a larger study which required more participants. Descriptive characteristics of the sample were calculated using IBM SPSS Statistics 22 and Sawtooth Software SSI Web (version 8.4.8) was used to calculate relative importances and part-worth utilities of the environmental attributes. Part-worth utilities and importances were calculated by Hierarchical Bayes estimation as recommended [41, 45], using dummy coding. Relative importance percentages indicate the maximum effect each included attribute (e.g. separation of cycle path and evenness of cycle path) has on participants’ preferences for a street/situation to cycle. Part-worth utilities are considered as the preferences for a particular level of an attribute (e.g. very uneven cycle path versus moderately uneven cycle path) and can be interpreted similar to regression coefficients in regression analyses [41]. Relative importances and part-worth utilities were calculated and 95% confidence intervals were constructed to compare relative importances and part-worth utilities. Relative importances and part-worth utilities within one attribute with non-overlapping 95% confidence intervals are significantly different from each other with α = 0.05. The fit of the conjoint model was presented by the Root LikeliHood (RLH) which ranges between 0 and 1. For a choice exercise with two alternatives, the RLH should be substantially larger than 0.5 [41]. Furthermore, to assess the validity of the model, the percentage of agreement between the choice predicted by the model and the actual choice of the participants in the two different fixed tasks was calculated. This represents for how many participants the choice predicted by the model corresponded to the actual choice of the participants. In order to investigate which physical environmental, social environmental and individual factors determine adolescents’ intention to actually cycle for transport in the preferred situation, logistic regression analyses were performed using R Studio version 3.1.0. For these analyses, three levels (school, participant and choice task) were taken into account.

## Results

### Sample characteristics

Participants who were not able to execute the choice tasks properly due to technical problems (*n* = 7) were removed from the dataset as were participants not completing the choice tasks (*n* = 33). This resulted in 973 complete cases. Test-retest reliability of the two fixed tasks resulted in a percentage agreement of 90.6% which corresponds to 91 participants not responding consistently. After exclusion of inconsistent responders, a final sample of 882 adolescents (87.1%) was used for data analyses.

Table 2 presents socio-demographic characteristics and transport data of the sample (*n* = 882). Mean age was 13.9 ± 1.6 years and 55.3% of the sample was male. Furthermore, 76.1% of adolescents lived in a semi-urban area and 78.7% had parents of higher SES. Approximately one fifth (19.0%) of adolescents did not cycle for transport in the last week. Among those who cycled, a median of 120 min cycling for transport in the last week was reported.

**Table 2:** Descriptive characteristics of the sample (*n* = 882)

Gender (% male)	55.3
Age (yrs; mean ± SD)	13.9 ± 1.6
Nationality (% Belgian)	97.2
Living environment (%)	
Rural area	10.0
Semi-urban area	76.1
Urban area	13.9
Socio-economic status (SES) (%)	
Lower SES (both parents completed only primary or secondary education)	21.3
Higher SES (at least one parent completed tertiary education)	78.7
Grade (%)	
1st year of secondary school	42.1
2nd year of secondary school	11.9
3rd year of secondary school	27.1
4th year of secondary school	18.9
Educational type^a^ (%)	
General studies	61.6
Technical studies	27.9
Occupational studies	10.5
Bicycle ownership (%)	96.6
No cycling for transport past week (%)	19.0
Cycling for transport among those who cycled in the past week (minutes/week; median)	120

^a^Main study disciplines available for secondary school students in Belgium, in which general studies prepare for college/university, technical studies have a more technical and practical approach, and occupational studies are more job specific

### Physical and social environmental preferences towards cycling for transport

**Fig. 3:**
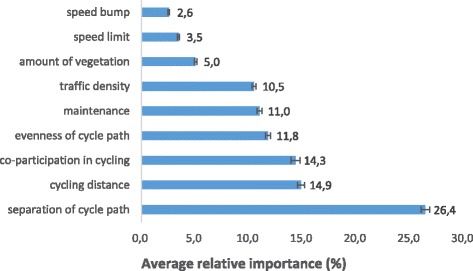
The relative importance and standard errors of physical micro-environmental factors, cycling distance and co-participation in cycling

Table 3 presents results on the relative importance of physical and social environmental factors on adolescents’ preferences towards cycling for transport. Separation of cycle path was the most important factor regarding adolescents’ preferences towards cycling for transport (see Fig. 3). This factor was chosen over cycling distance and co-participation in cycling, for which the importances did not differ significantly from each other. Evenness of cycle path was the fourth most important factor followed by maintenance and traffic density, for which the importances did not differ significantly from each other. Consecutively, importances were significantly lower for amount of vegetation, speed limit and speed bump.

**Table 3:** Results on physical and social environmental, and individual factors associated with adolescents’ preferences and intention to cycle for transport

	Environmental preferences		Associations with intention	
	Relative importances	Part-worth utilities	Wald test Chi^2^	OR (95% CI)
	(%, 95% CI)	(95% CI)		
Separation of cycle path	26.4 (25.7; 27.2)		54.0***	
No cycle path		reference category		reference category
Cycle path separated from traffic with lines, not separated from walking path (advisory cycle path)		4.9 (4.7; 5.0)		2.1 (1.4; 3.0)
Cycle path separated from traffic with a curb, not separated from walking path		5.5 (5.4; 5.7)		3.4 (2.3; 4.9)
Cycle path separated from traffic with a hedge, not separated from walking path		7.1 (7.0; 7.2)		3.0 (2.1; 4.3)
Cycle path separated from traffic with a curb, separated from walking path by colour		6.2 (6.1; 6.3)		2.9 (2.0; 4.2)
Cycle path separated from traffic with a hedge, Separated from walking path by colour		6.5 (6.3; 6.8)		3.1 (2.1; 4.5)
**Cycling distance**	14.9 (14.2; 15.5)		12.7*	
15 min		reference category		reference category
14 min		0.3 (0.1; 0.5)		0.8 (0.6; 1.1)
13 min		1.3 (1.1; 1.4)		0.8 (0.6; 1.1)
12 min		1.7 (1.6; 1.8)		1.1 (0.8; 1.5)
11 min		2.3 (2.2; 2.4)		1.1 (0.8; 1.6)
10 min		2.6 (2.4; 2.9)		1.3 (0.9; 1.8)
Co-participation in cycling	14.4 (13.5; 15.2)		24.9***	
Alone		reference category		reference category
With a friend		3.4 (3.2; 3.6)		1.7 (1.4; 2.0)
Evenness of cycle path	11.8 (11.4; 12.3)		68.8***	
Very uneven		reference category		reference category
Moderately uneven		1.1 (1.0, 1.2)		1.3 (1.0; 1.7)
Even		3.1 (3.0; 3.3)		2.6 (2.1; 3.4)
Maintenance	11.0 (10.6, 11.4)		26.8***	
Poor upkeep (much graffiti and litter)		reference category		reference category
Moderate upkeep (a bit of graffiti and litter)		1.9 (1.8; 2.0)		1.3 (1.0; 1.6)
Good upkeep (no graffiti or litter)		2.8 (2.6; 2.9)		1.9 (1.5; 2.4)
Traffic density	10.5 (10.1, 10.9)		15.1***	
4 cars + truck		reference category		reference category
3 cars		1.7 (1.6; 1.8)		1.2 (0.9; 1.5)
1 car		2.6 (2.5; 2.7)		1.6 (1.3; 2.0)
Amount of vegetation	5.0 (4.9, 5.2)		1.0	
No trees		reference category		reference category
Two trees		0.5 (0.4; 0.5)		1.1 (0.9; 1.3)
Four trees		0.4 (0.3; 0.5)		1.1 (0.9; 1.4)
Speed limit	3.5 (3.3, 3.6)		2.4	
50 km/h		reference category		reference category
30 km/h		0.6 (0.6; 0.7)		1.2 (1.0; 1.4)
Speed bump	2.6 (2.4, 2.7)		1.5	
Absent		reference category		reference category
Present		−0.1 (−0.1; 0.0)		0.9 (0.7; 1.1)
Gender			3.7^t^	
Male				reference category
Female				0.5 (0.3; 1.0)
SES parents			0.3	
Lower SES				reference category
Higher SES				1.2 (0.5; 2.8)
Age			2.8^t^	1.2 (1.0; 1.5)
Social modelling			17.7***	2.7 (1.7; 4.3)
Social support			1.4	1.3 (0.9; 1.8)
Social norms			2.0	1.3 (0.9; 1.8)
Self-efficacy			21.3***	1.9 (1.5; 2.6)
RLH	0.9			
Agreement model prediction – fixed task 1 (%)^a^	96.8			
Agreement model prediction – fixed task 2 (%)^a^	78.6			

*CI* confidence interval, *OR* odds ratio, *RLH* Root LikeliHood

***p < 0.001; **p < 0.01; *p < 0.05; ^t^ *p* < 0.1

^a^This represents for how many participants the choice predicted by the model corresponds to the actual choice of the participants

**Fig. 4:**
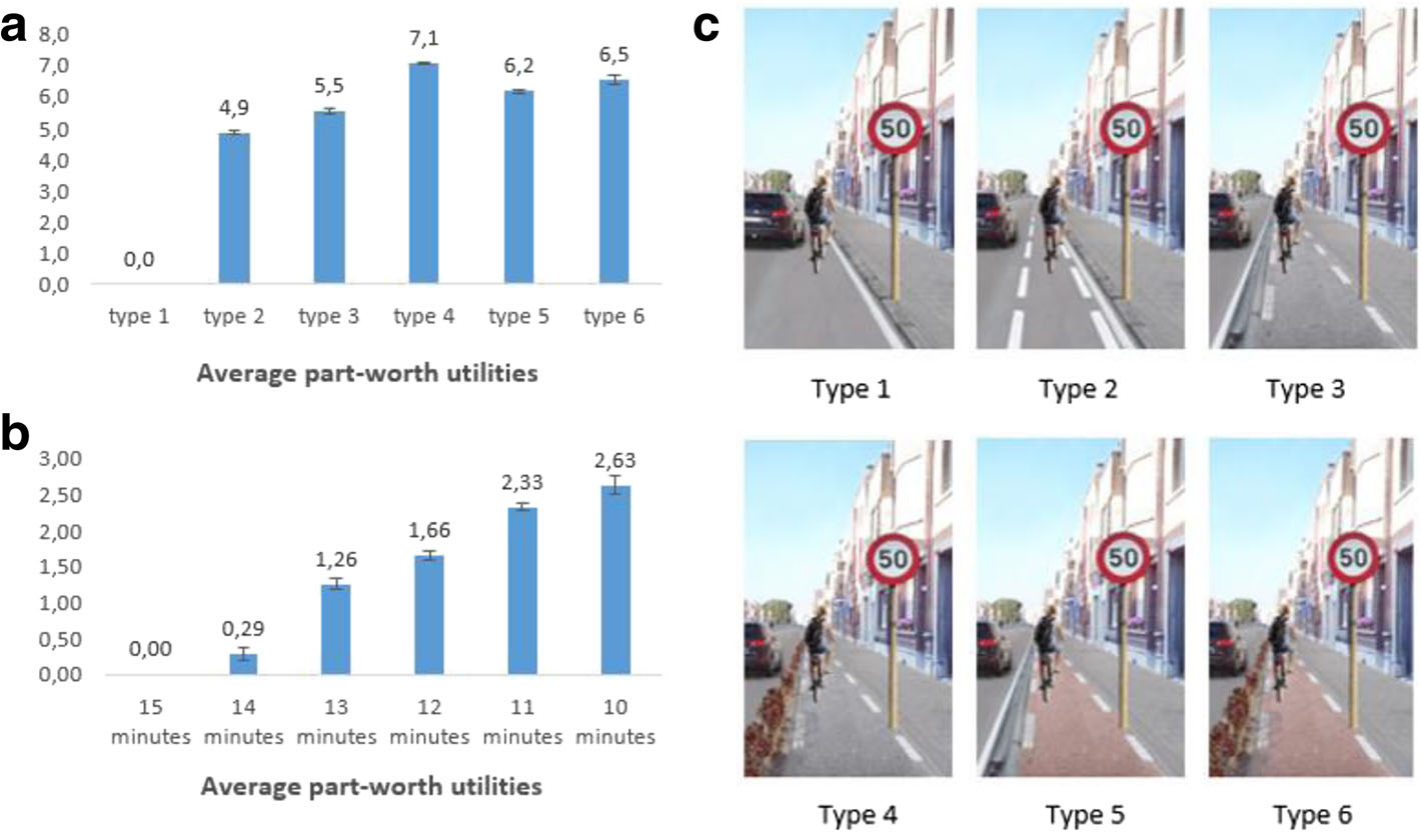
Part-worth utilities/preferences within separation of cycle path (**a**) and cycling distance (**b**). Section (**c**) visually shows the different levels for separation of cycle path

Within each physical and social environmental factor, clear preferences for a specific level were observed, except for speed bump (see Table 3; part-worth utilities). Within separation of cycle path, the presence of any separation of cycle path was preferred to no cycle path at all (part-worth utilities of all separations of cycle path differed significantly from the reference category, i.e. having no cycle path (type 1)). Adolescents preferred to cycle on a cycle path separated from traffic with a hedge, but not separated from the walking path (see Fig. 4 – type 4) over all other separations of cycle path. Within cycling distance, a shorter distance was preferred over longer distances, but the importance of a 10-min cycling route did not differ significantly from the importance of an 11-min cycling route. For co-participation in cycling, evenness of the cycle path, maintenance, traffic density and speed limit, the anticipated best level was preferred (i.e. cycling with a friend, even cycle path, good maintenance, one car and 30 km/h). For amount of vegetation, the preference for four or two trees did not differ significantly, but these two levels were preferred over no trees.

### Physical environmental, social environmental and individual factors associated with intention to actually cycle for transport

Results on associations between physical environmental, social environmental and individual factors, and intention to actually cycle to a friends’ house are presented in Table 3 (Wald test Chi^2^ and OR). In 79.0% of the choice tasks participants indicated that they would actually cycle in the preferred situation. Results showed that separation of cycle path, evenness of cycle path, maintenance, traffic density, cycling distance, co-participation in cycling, social modelling for actual cycling in real life and self-efficacy towards cycling were significantly associated with intention to actually cycle for transport. For each separation of cycle path, the odds of adolescents indicating that they would actually cycle were 110–240% higher compared to when no cycling path was present. For cycling distance, the odds of adolescents indicating that they would actually cycle were 40% lower when distance increased to 14 or 13 min compared to 10 min (results are not shown in Table 3). Regarding co-participation in cycling, the odds of adolescents indicating that they would actually cycle were 70% higher when they could cycle together with a friend compared to cycling alone. Furthermore, for a moderately uneven cycle path adolescents had 30% higher odds of indicating that they would actually cycle and for an even cycle path they had 160% higher odds of indicating that they would actually cycle compared to when a very uneven cycle path was present. Also for maintenance, an improvement by one or two levels resulted in 30% and 90% higher odds to indicate that they would actually cycle, respectively. For traffic density, an improvement by one or two levels resulted in 20% and 60% higher odds to indicate that they would actually cycle, respectively. In addition, the odds of indicating that they would actually cycle were nearly three times higher when perceived social modelling for actual cycling in real life was one unit higher. Finally, the odds of adolescents indicating that they would actually cycle were 90% higher when self-efficacy for cycling increased by one unit.

## Discussion

The present study examined the relative importance of physical and social environmental factors regarding adolescents’ preferences towards a situation to cycle for transport. In addition, this study aimed to examine the influence of individual, physical and social environmental factors on adolescents’ intention to actually cycle for transport in the preferred situation. This was the first experimental study using manipulated photographs to investigate adolescents’ preferences towards cycling for transport and their intention to actually cycle in preferred situations. Adolescents’ preference to cycle for transport in a particular situation was predominantly determined by separation of cycle path. In addition, separation of cycle path also had a significant influence on their intention to actually cycle for transport in the preferred situation. This is consistent with findings from previous studies among children and adults [32, 34], although it should be mentioned that the importance of separation of cycle path was less distinct among adolescents compared to other age groups. Based on results of previous studies among adolescents [19, 22, 35–37], it could be expected that cycling distance and co-participation in cycling would be more important for adolescents’ cycling for transport than physical micro-environmental factors. However, this study showed that type of cycle path was more important for adolescents in order to cycle for transport than cycling distance and co-participation in cycling. These findings justify investment by local governments in well-separated cycling infrastructure. Clear preferences were observed for a cycle path separated from traffic with a hedge over separations by a curb or marked line, which is also in line with findings among children and adults [31, 32, 34].

Previous studies emphasized the importance of cycling distance and the social environment on adolescents’ cycling for transport [8, 15, 19, 22, 37, 38]. The present study confirmed the importance of cycling distance and co-participation in cycling since these were the second most important factors influencing adolescents’ preferences towards a situation to cycle for transport and these factors were also significantly associated with intention to actual cycling. However, results showed that these factors were less important than separation of the cycle path, although, based on results of previous qualitative and quantitative studies among adolescents, one would expect the social environment to be more important [22, 37]. Consistent with previous studies [8, 15, 19], clear preferences were observed for shorter cycling distances. However, in this experiment the difference between the shortest (10 min) and the longest (15 min) cycling route was relatively small. Since distance is a factor which is very difficult to change, the best option probably is to influence adolescents’ perception of what is ‘a long cycling distance’. However, other studies are needed to explore whether this perception can be influenced and how it can be done. Furthermore, the influence of the social environment should not be underestimated since being able to cycle together with a friend was of significant importance for adolescents’ preferences and intention to actually cycle for transport. In addition, social modelling proved to be positively related to adolescents’ active transport levels in previous studies [6, 22, 46] and the present study confirmed that perceiving more social modelling for cycling in real life was positively related to adolescents’ intention to actually cycle for transport in the presented environments. This emphasizes that interventions which focus on improving the physical environment to promote cycling for transport among adolescents might benefit from also involving the social environment in order to make adolescents cycle in real life.

Furthermore, adolescents’ preference for a particular cycling situation was less strongly but considerably influenced by evenness of the cycle path and general maintenance of the street. These factors were also significantly related to adolescents’ intention to actually cycle for transport in the preferred situation. Results of the present study showed that traffic safety issues such as traffic density, speed limit and speed bump are of minor importance for adolescents’ cycling for transport, although separation of cycle path, which is also related to traffic safety, was found to be the most important factor for adolescents’ preferences towards cycling for transport. Previous studies collecting qualitative information on transport mode choice among adolescents found that traffic safety is not a major concern among adolescents [8, 37]. When a well-separated cycle path is provided, micro-environmental factors related to comfort and aesthetics showed to be more important than other micro-environmental factors related to traffic safety. Changes in the built environment are likely to have an impact on different age groups living in that area [47]. Previous research among children and their parents also showed that general maintenance of the street seemed to be of considerable importance to increase the supportiveness of a street for cycling for transport [32]. Maintenance of the street may not only be related to aesthetics, but is also potentially related to feelings of safety from crime. Physical disorder (such as litter and graffiti) present visual cues that can have a negative impact on perceived safety from crime among youth and their parents [48]. This, in turn, may affect a street’s appeal for cycling. A previous study investigating the association between personal safety and walking for transport found that a neighbourhood which supports walking is also a place where residents feel safer [49]. This study stated that a greater investment in maintenance programs may increase residents’ perceived safety from crime. As a result, this may encourage active transport among children and adolescents. However, among 45–65 year olds, maintenance seems to be less important than traffic safety issues and evenness of the cycle path [34]. Although evenness of the cycle path was the second most important physical micro-environmental variable for adolescents, previous studies have shown it was less important for children and adults for which traffic density and speed limit were more important [32, 34]. These results need to be taken into account when implementing adaptations to the physical environment in order to encourage cycling across the entire population. Amount of vegetation and presence of a speed bump were the least important factors among adolescents, as was the case among children and their parents [32] and 45–65 year old adults [34]. Local authorities should therefore give priority to investments in important factors, such as the provision of a cycle path which is well-separated from motorised traffic.

### Practical implications

Based on our findings, some recommendations for policy and practice can be formulated. As resources of local governments are limited, they should be applied efficiently. In order to stimulate cycling among adolescents as well as among other age groups, first, it may be important to invest in cycling infrastructure that is physically separated from motorized traffic, preferably by a hedge or other physical separation. A cycle path that is completely separated from motorized traffic will probably be even better than a separation by a hedge, but this was not investigated in the present study. However, for local authorities with insufficient resources and in neighbourhoods with limited space for cycling infrastructure, even small improvements in cycling infrastructure such as a curb or marked line to separate cyclists from motorized traffic may potentially contribute to higher cycling rates. Other changes in the physical micro-environment that may stimulate cycling for transport are improvements in evenness of the cycle path and investing in well-maintained streets. In addition, since co-participation in cycling also seemed to be important for adolescents, local authorities should thus provide well-separated cycle paths which are, preferably, also wide enough in order that people can cycle next to each other in a safe and comfortable way when (re)designing neighbourhoods. Finally, the present study confirmed the importance of a short cycling distance for adolescents. Although cycling distance is not really modifiable, local authorities could provide shortcuts which are only accessible for pedestrians and cyclists when developing new neighbourhoods.

## Strengths and limitations

The most important strength of the current study was the experimental use of manipulated photographs to investigate the importance of physical and social environmental factors and their corresponding levels for adolescents’ preferences towards a situation to cycle for transport and their intention to actually cycle. This method tackled limitations of previous questionnaire-based studies. The use of an experimental design enabled us to investigate causal relationships between physical and social environmental factors, and adolescents’ preferences and intention towards cycling for transport. Furthermore, the present study investigated the influence of cycling distance and co-participation in cycling on adolescents’ choice to cycle for transport, which was missing in previous studies using manipulated photographs. In addition, next to investigating adolescents’ preferred setting to cycle for transport, the present study also investigated adolescents’ intention to actually cycle for transport in that situation.

Despite the benefits of using manipulated photographs, the most important limitation of this study is that this method did not enable to investigate associations with actual participation in cycling for transport. Conducting natural experiments may be a potential strategy to examine the effect of real changes in physical micro-environmental factors on adolescents’ cycling for transport, although introducing structural changes in real environments is very expensive and time-consuming. Observational studies may also provide some insights into the role of the physical environment on adolescents’ cycling for transport. However, manipulated photographs enabled us to simulate changes under controlled conditions. Nevertheless, factors such as noise and car exhausts, but also busy crossings cannot be captured in a photograph. Therefore, results cannot be generalized to other street settings (such as busy crossings) which emphasizes the need to introduce more diverse street settings in future studies. Studies using virtual reality might bridge the gap between manipulated photographs and real life situations. Furthermore, the authors acknowledge that some attributes may be more clearly visible due to the composition of the photographs. Type of cycle path, for example, was presented centrally in each photograph which may have influenced the results. However, in two previously conducted pilot studies [27, 28], participants were asked to sort manipulated photographs of a street from least to most inviting to walk or cycle for transport. Participants also provided qualitative data on how they sorted the streets. Results of these studies showed that attributes which were less clearly visible in the photographs also seemed to be of importance regarding invitingness of a street to walk or cycle for transport. Therefore, the composition of the photographs probably had only a minimal effect on the results of the present study. Finally, by also assessing participants’ intention to cycle for transport in the situation they did not prefer, correlates of intention could be derived from broader situations.

## Conclusions

Results of this experimental study using manipulated photographs showed that the physical environment seems to be important for adolescents’ cycling for transport, although based on results of previous qualitative and quantitative studies one would expect the social environment to be more important. This study showed that local authorities should give priority to the provision of cycle paths which are well-separated from motorised traffic when aiming to promote cycling for transport among adolescents. Adolescents seem to prefer cycle paths separated from motorised traffic by a hedge, followed by separations by means of a curb or marked line. The present study was able to confirm findings of previous studies that cycling distance and co-participation of friends are important factors for adolescents’ cycling for transport, but showed that separation of the cycle path is more important. Other changes in the micro-environment that may enhance cycling for transport among adolescents, though to a lesser extent than cycling distance and co-participation, include the provision of an even cycle path and investments in a well-maintained environment. If natural experiments or observational studies can confirm findings of this study in real life settings, local authorities can be informed about which changes in the environment should be prioritised when trying to increase adolescents’ cycling for transport.
